# The C-Terminal Domain of Nef^mut^ Is Dispensable for the CD8^+^ T Cell Immunogenicity of In Vivo Engineered Extracellular Vesicles

**DOI:** 10.3390/vaccines9040373

**Published:** 2021-04-12

**Authors:** Chiara Chiozzini, Francesco Manfredi, Flavia Ferrantelli, Patrizia Leone, Andrea Giovannelli, Eleonora Olivetta, Maurizio Federico

**Affiliations:** 1 National Center for Global Health, Istituto Superiore di Sanità, Viale Regina Elena 299, 00161 Rome, Italy; chiara.chiozzini@iss.it (C.C.); francesco.manfredi@iss.it (F.M.); flavia.ferrantelli@iss.it (F.F.); patrizia.leone@iss.it (P.L.); eleonora.olivetta@iss.it (E.O.); 2 National Center for Animal Experimentation and Welfare, Istituto Superiore di Sanità, Viale Regina Elena 299, 00161 Rome, Italy; andrea.giovannelli@iss.it

**Keywords:** extracellular vesicles, HIV-1 Nef, DNA immunization, CD8^+^ T cell immunity, HPV vaccines, SARS-CoV-2 vaccines

## Abstract

Intramuscular injection of DNA vectors expressing the extracellular vesicle (EV)-anchoring protein Nef^mut^ fused at its C-terminus to viral and tumor antigens elicit a potent, effective, and anti-tolerogenic CD8^+^ T cell immunity against the heterologous antigen. The immune response is induced through the production of EVs incorporating Nef^mut^-derivatives released by muscle cells. In the perspective of a possible translation into the clinic of the Nef^mut^-based vaccine platform, we aimed at increasing its safety profile by identifying the minimal part of Nef^mut^ retaining the EV-anchoring protein property. We found that a C-terminal deletion of 29-amino acids did not affect the ability of Nef^mut^ to associate with EVs. The EV-anchoring function was also preserved when antigens from both HPV16 (i.e., E6 and E7) and SARS-CoV-2 (i.e., S1 and S2) were fused to its C-terminus. Most important, the Nef^mut^ C-terminal deletion did not affect levels, quality, and diffusion at distal sites of the antigen-specific CD8^+^ T immunity. We concluded that the C-terminal Nef^mut^ truncation does not influence stability, EV-anchoring, and CD8^+^ T cell immunogenicity of the fused antigen. Hence, the C-terminal deleted Nef^mut^ may represent a safer alternative to the full-length isoform for vaccines in humans.

## 1. Introduction

Healthy cells constitutively release nanovesicles which are identified as microvesicles (50–1000 nm diameter), and exosomes (50–200 nm), together referred to as EVs [1]. Microvesicles (also identified as ectosomes) are shed by the plasma membrane, whereas exosomes are released after inward invagination of endosome membranes, formation of intraluminal vesicles, and release. EVs are an important means of intercellular communication because of their ability to transport DNAs, RNAs, proteins, and lipids from the producer cell to the recipient one [2].

Nef^mut^ is a Human Immunodeficiency Virus-1 protein mutated in ^G^3^C^, ^V^153^L^, and ^E^177^G^ amino acids. It is a protein mutant defective for all anti-cellular Nef functions, including CD4 and MHC Class I down-regulation, increased HIV-1 infectivity, and p21 activated kinase (PAK)-2 activation [3]. We previously described the high efficiency of uploading HIV-1 Nef^mut^ into EVs released by multiple cell types [4], which remains unchanged when a foreign protein is fused to its C-terminus [5,6,7]. When DNA vectors expressing Nef^mut^-based fusion proteins are intramuscularly (i.m.) injected in mice, significant quantities of the fusion proteins are packed into EVs. These in vivo engineered EVs can freely circulate into the body, thereby being internalized by antigen-presenting cells (APCs), which cross-present EV contents to activate antigen-specific CD8^+^ T cells. These events result in the induction of potent antigen-specific CD8^+^ T cytotoxic lymphocyte (CTL) responses [6,7,8,9]. Both effectiveness and flexibility of this vaccine platform have been demonstrated with an array of viral products of various origins and sizes, including but not limited to Human Papillomavirus (HPV)16-E6 and -E7 [8,10]; Severe acute Respiratory Syndrome Coronavirus (SARS-CoV)-2 S1, S2, M, and N [11]; HIV-1 Gag p17, Gag p24 and Tat [12]; Ebola Virus VP24, VP40 and NP [7]; Hepatitis B Virus Core [12]; Hepatitis C Virus NS3, West Nile Virus NS3, and Crimean-Congo Hemorrhagic Fever NP [5,7].

To translate into the clinic the Nef^mut^-based vaccine platform, we attempted to maximize its intrinsic safety profile by identifying the minimum part of Nef^mut^ retaining both the EV-anchoring protein and immunogenicity properties. Investigations were carried out by fusing Nef^mut^-derivatives with antigens from viruses recognizing different pathogenic effects, i.e., HPV16, whose infection can induce tumors, and the acute respiratory disease-inducing SARS-CoV-2.

Pre-clinical studies that we had already carried out provided evidence that the Nef^mut^-based CTL vaccine platform can act as an effective therapeutic intervention against HPV16-related and other malignancies [6,9]. The benefits expected from a therapeutic cancer vaccine are the possibility to induce a de novo antitumor immunity, as well as widen both potency and breadth of pre-existing immunity [13,14]. On the other hand, both experimental and clinical evidence supported the idea that a SARS-CoV-2-specific CD8^+^ T cell immunity can be instrumental to mitigate the symptoms related to the viral spread in both upper and lower airways [15,16]. Data from experimental infections in rhesus macaques indicated that the virus-specific CD8^+^ T cell immunity is critical to protect the animals from virus re-challenge after the rapid decay of neutralizing antibodies [17]. Consistently, the induction of antiviral CD8^+^ T cells was associated with a strongly reduced severity of the disease in humans [18]. Furthermore, the demonstrated ability of SARS-CoV-2 to spread through cell-to-cell contact [19] implies the need to induce a robust cellular immunity to contain and clear the virus. In this context, the development of novel preventive strategies focused on the induction of anti-SARS-CoV-2 CD8^+^ T cell immunity should be pursued. We recently demonstrated that the Nef^mut^-based technology is functional to induce CD8^+^ T cell immunity against different SARS-CoV-2 antigens [11].

To increase the safety profile of the Nef^mut^-based technology, we found that both EV-incorporation efficiency and immunogenicity of foreign antigens are conserved in the presence of a C-terminal 29 amino acid deletion of Nef^mut^.

## 2. Materials and Methods

### 2.1. DNA Vector Synthesis

The pTargeT (Invitrogen, Thermo Fisher Scientific, Waltham, MA, USA)-Nef^mut^ and -Nef^mut^fusion vectors were already described [6,12]. The pTargeT-Nef^mut^/E6 vector comprised an E6 open reading frame (ORF), which was codon-optimized through an ad hoc algorithm provided by the Codon Optimization On-Line (COOL) service (https://cool.syncti.org, accessed on 15 January 2018), which introduced 134 base substitutions. In addition, we included the ^G^130^V^ amino acid substitution, which generated a loss-of-function of E6 by hindering its interaction with the p53 cell protein partner [20]. The E6 ORF was inserted in *Apa* I/*Sal* I sites of the pTargeT-Nef^mut^fusion vector. In the pTargeT-Nef^mut^/E7 vector, the E7 ORF was codon-optimized through the introduction of 64 base substitutions as described by Cid-Arregui and coll. [21]. Furthermore, the protein was detoxified through the insertion of three amino acid substitutions, namely three glycines at positions 21, 24, and 26, within the retinoblastoma protein (pRB) binding site. In this way, the E7-specific immortalizing activity was abrogated [21].

**Figure 1:**
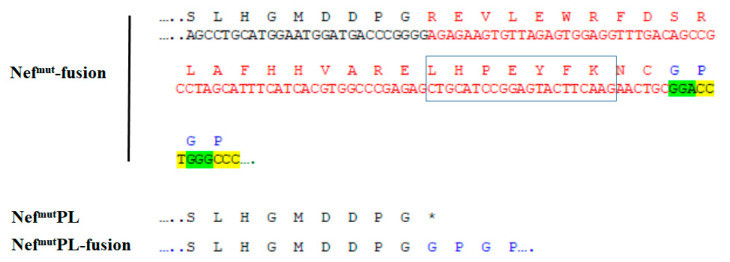
Nef C-terminal sequences in both Nef^mut^PL and Nef^mut^PLfusion DNA vectors. On the top, both nucleotide and amino acid sequences at the C-terminus of Nef^mut^ ORF in the context of both pTargeT- and pVAX1-Nef^mut^fusion vectors are reported. Sequences just before the truncation are in blue, sequences of the 29-amino acid region deleted in the Nef^mut^PL ORF are in red, and the linker sequence is in green/yellow. The CRM domain is highlighted. At the bottom, amino acid sequences of Nef C-terminal regions in both Nef^mut^PL and Nef^mut^PLfusion vectors are reported. In the latter case, the position of the GPGP linker is indicated. * marks the stop codon.

The C-terminal truncated Nef^mut^ (referred to as Nef^mut^PL) was inserted in the pTargeT vector by digesting the pTargeT-Nef^mut^ vector with *Sma* I, which cuts just downstream to the most C-terminal typical Nef^mut^ mutation (i.e., ^E^177^G^), as well as at the 3′ end of vector polylinker. The subsequent re-ligation generated a C-terminal 29 amino acid deletion with the generation of a stop codon just downstream the *Sma* I restriction site (Figure 1).

To obtain the pTargeT-Nef^mut^-PL-based DNA vectors, an intermediate construct referred to as Nef^mut^PLfusion was constructed. In detail, the Nef^mut^PL ORF from pTargeT-Nef^mut^PL was PCR amplified using a forward primer tagged with a *Nhe* I restriction site, and a reverse primer including an *Apa* I site together with an overlapping sequence for a GPGP linker. The PCR product was then inserted into the corresponding restriction sites of the pTargeT vector. In this way, the insertion in the unique *Apa* I restriction site of downstream ORFs resulted in an in-frame sequence. To obtain the pTarget-Nef^mut^PL/E6 and pTarget-Nef^mut^PL/E7 vectors, synthesis and cloning strategy were identical to those described for the DNA vectors expressing full-length Nef^mut^.

Both Nef^mut^/SARS-CoV-2-based fusion proteins were cloned into the pVAX1 plasmid (Thermo Fisher) as already described [11]. Both pVAX1-Nef^mut^ and pVAX1-Nef^mut^PL vectors were obtained by inserting the respective ORFs in *Nhe* I and *Eco R*I sites of the vector polylinker. To obtain pVAX1 vectors expressing Nef^mut^PL fused with either SARS-CoV-2 S1 or S2 ORFs, an intermediate construct referred to as pVAX1-Nef^mut^PLfusion was obtained. To this aim, the Nef^mut^PL ORF from the pTargeT-Nef^mut^PL vector was PCR amplified using a forward primer tagged with a *Nhe* I restriction site, and a reverse primer, including an *Apa* I and the overlapping sequence for a GPGP linker. The amplification product was then inserted in the corresponding sites of the pVAX1 vector. In this way, the downstream insertion of S1 or S2 ORFs in *Apa* I and *Pme* I restriction sites resulted in in-frame sequences, and, upon translation, in Nef^mut^PL-based fusion proteins. Eurofins Genomics and Explora Biotech carried out gene synthesis.

### 2.2. Cell Cultures and Transfection

Human embryonic kidney (HEK)293T cells (ATCC, CRL-11268) were grown in DMEM (Gibco, Thermo Fisher) plus 10% heat-inactivated fetal calf serum (FCS, Gibco, Thermo Fisher). Transfection assays were performed using Lipofectamine 2000 (Invitrogen, Thermo Fisher Scientific).

### 2.3. EV Isolation

Cells transfected with vectors expressing the Nef^mut^-based fusion proteins were washed 24 h later and reseeded in a medium supplemented with EV-deprived FCS. The supernatants were harvested from 48 to 72 h after transfection. EVs were recovered through differential centrifugations [22] by centrifuging supernatants at 500× *g* for 10 min, and then at 10,000× *g* for 30 min. Supernatants were harvested, filtered with 0.22 µm pore size filters, and ultracentrifuged at 70,000× *g* for l h. Pelleted vesicles were resuspended in 1× PBS, and ultracentrifuged again at 70,000× *g* for 1 h. Afterward, pellets containing EVs were resuspended in 1:100 of the initial volume.

### 2.4. Western Blot Analysis

Western blot analyses of both cell lysates and EVs were carried out after resolving samples in 10% sodium dodecyl sulfate-polyacrylamide gel electrophoresis (SDS-PAGE). In brief, the analysis on cell lysates was performed by washing cells twice with 1× PBS (pH 7.4) and lysing them with 1 × SDS-PAGE sample buffer. Samples were resolved by SDS-PAGE and transferred by electroblotting on a 0.45 μM pore size nitrocellulose membrane (GE Healthcare Europe GmbH, Milan, Italy) overnight using a Bio-Rad (Hercules, CA, USA) Trans-Blot. For western blot analysis of EVs, they were lysed and analyzed as described for cell lysates. For immunoassays, membranes were blocked with 5% non-fat dry milk in PBS containing 0.1% Triton X-100 for 1 h at room temperature, then incubated overnight at 4 °C with specific antibodies diluted in PBS containing 0.1% Triton X-100. Filters were revealed using 1:1000-diluted sheep anti-Nef antiserum ARP 444 (MHRC, London, UK), 1:500-diluted anti-β-actin AC-74 mAb from Sigma (St. Louis, MI, USA), and 1:500 diluted anti-Alix H-270 polyclonal Abs from Santa Cruz (Dallas, TX, USA).

### 2.5. Mice Immunization

Both 6-weeks old C57 Bl/6 and for Nef^mut^/S2 immunizations (in view of the lack of already characterized H2^b^ immunodominant S2 epitopes), Balb/c female mice were obtained from Charles River (Calco, Italy). They were hosted at the Central Animal Facility of the Istituto Superiore di Sanità (ISS), as approved by the Italian Ministry of Health, authorization n. 565/2020 released on 3 June 2020. Preparations of the DNA vector were diluted in 30 µL of sterile 0.9% saline solution. Both the quality and quantity of the DNA preparations were checked by 260/280 nm absorbance and electrophoresis assays. Each inoculum volume was injected into both quadriceps. Mice were anesthetized with isoflurane as prescribed in the Ministry authorization. Immediately after inoculation, mice underwent electroporation at the site of injection through the Agilpulse BTX (Holliston, MA, USA) device using a 4-needle array 4 mm gap, 5 mm needle length, with the following parameters: 1 pulse of 450 V for 50 µs; 0.2 ms interval; 1 pulse of 450 V for 50 µs; 50 ms interval; 8 pulses of 110 V for 10 ms with 20 ms intervals. The same procedure was repeated for both quadriceps of each mouse. Immunizations were repeated after 14 days. Fourteen days after the second immunization, mice were sacrificed by either cervical dislocation or CO_2_ inhalation.

### 2.6. Cell Isolation from Immunized Mice

Spleens were explanted by qualified personnel of the ISS Central Animal Facility and placed into 2 mL Eppendorf tubes filled with 1 mL of RPMI 1640 (Gibco), 50 µM 2-mercaptoethanol (Sigma). Splenocytes were extracted as already detailed [11]. The procedures for bronchoalveolar lavages were carried out as previously described [23,24]. Total lavage volume was approximately 2.5 mL/mouse. Cells were recovered by centrifugation, resuspended in cell culture medium, and counted.

### 2.7. IFN-γ EliSpot Analysis

A total of 2.5 × 10^5^ live cells was seeded in triplicate EliSpot microwells (Millipore, Burlington, MA, USA) pre-coated with the AN18 mAb against mouse IFN-γ (Mabtech, Nacka Strand, Sweden) in RPMI 1640 (Gibco), 10% FCS, 50 µM 2-mercaptoethanol (Sigma) for 16 h in the presence of 5 µg/mL of the following CD8-specific peptides: E6 (H2-K^b^): 18–26: KLPQLCTEL [25]; 50–57: YDFAFRDL [25]; 109–117: RCINCQKPL [26]; 127–135: DKKQRFMNI [25]. HPV-16 E7 (H2-K^b^): 49–57 RAHYNIVTF [25]; 67–75 LCVQSTHVD [26]. SARS-CoV-2 S1 (H2-K^b^): 525–531 VNFNFNGL [27]; SARS-CoV-2 S2 (H2-K^d^): 1079–1089 PAICHDGKAH [28]. As a negative control, 5 µg/mL of either H2-K^b^ or H2-K^d^-binding peptides were used. More than 70% pure preparations of the peptides were obtained from both UFPeptides, Ferrara, Italy, and JPT, Berlin, Germany. For cell activation control, cultures were treated with 10 ng/mL phorbol 12-myristate 13-acetate (PMA, Sigma) plus 500 ng/mL of ionomycin (Sigma). After 16 h, the cultures were removed, and wells incubated with 100 µL of 1 µg/mL of the R4-6A2 biotinylated anti-IFN-γ (Mabtech) for 2 h at r.t. Wells were then washed and treated for 1 h at r.t. with 1:1000 diluted streptavidine-ALP preparations from Mabtech. After washing, spots were developed by adding 100 µL/well of SigmaFast BCIP/NBT. The spot-forming cells were finally analyzed and counted using an AELVIS EliSpot reader (Hannover, Germany).

### 2.8. Intracellular Cytokine Staining (ICS)

Splenocytes were seeded at 2 × 10^6^/mL in RPMI medium, 10% FCS, 50 µM 2-mercaptoethanol (Sigma), and 1 µg/mL brefeldin A (BD Biosciences, Franklin Lakes, NJ, USA). Control conditions were carried out either by adding 10 ng/mL PMA (Sigma) and 1 µg/mL ionomycin (Sigma) or with unrelated peptides. After 16 h, cultures were stained with 1 µL of LIVE/DEAD Fixable Aqua Dead Cell reagent (Invitrogen, ThermoFisher) in 1 mL of PBS for 30 min at 4 °C and washed twice with 500 µL of PBS. To minimize nonspecific staining, cells were pre-incubated with 0.5 µg of Fc blocking mAbs (i.e., anti-CD16/CD32 antibodies, Invitrogen/eBioscience, Thermo Fisher, Waltham, MA) in 100 µL of PBS with 2% FCS for 15 min at 4 °C. For the detection of cell surface markers, cells were stained with 2 µL of the following Abs: FITC conjugated anti-mouse CD3, APC-Cy7 conjugated anti-mouse CD8a, and PerCP conjugated anti-mouse CD4 (BD Biosciences) and incubated for 1 h at 4 °C. After washing, cells were permeabilized and fixed through the Cytofix/Cytoperm kit (BD Biosciences) as per the manufacturer’s recommendations and stained for 1 h at 4 °C with 2 µL of the following Abs: PE-Cy7 conjugated anti-mouse IFN-γ (BD Biosciences), PE-conjugated anti-mouse IL-2 (Invitrogen eBioscience), and BV421 anti-mouse TNF-α (BD Biosciences) in a total of 100 µL of 1× Perm/Wash Buffer (BD Biosciences). After two washes, cells were fixed in 200 µL of 1× PBS/formaldehyde (2% *v*/*v*). Samples were then assessed by a Gallios flow cytometer and analyzed using Kaluza software (Beckman Coulter, Brea, CA, USA).

Gating strategy was as follows: live cells as detected by Aqua LIVE/DEAD Dye vs. FSC-A, singlet cells from FSC-A vs. FSC-H (singlet 1) and SSC-A vs SSC-W (singlet 2), CD3 positive cells from CD3 (FITC) vs. SSC-A, CD8 or CD4 positive cells from CD8 (APC-Cy7) vs. CD4 (PerCP). The CD8^+^ cell population was gated against APC-Cy7, PE, and BV421 to observe changes in IFN-γ, IL-2, and TNF-α production, respectively. Boolean gates were created to determine any cytokine co-expression pattern.

### 2.9. Statistical Analysis

When appropriate, data are presented as mean + standard deviation (SD). In some instances, the Mann-Whitney U test was used. *p* < 0.05 was considered significant.

## 3. Results

### 3.1. Design and EV-Uploading of a C-Terminal Truncated Nef^mut^

Removing unnecessary sequences from the Nef^mut^ EV-anchoring protein can increase the safety profile of the Nef^mut^ CTL vaccine platform. We tried to identify the shortest Nef^mut^ amino acid sequence retaining the ability to incorporate into EVs at high levels.

HIV-1 Nef is a 206 to 210 amino acid long protein recognizing an extended structured N-moiety, and an unstructured C-terminal flexible region [29]. To leave untouched the Nef^mut^ secondary structure, which is assumed to be critical for the EV-uploading activity, isoforms deleted of part of the unstructured C-terminus were considered. Another relevant constraint was represented by the most C-terminal unique amino acid substitution of Nef^mut^ (i.e., ^E^177^G^) whose presence is mandatory to preserve the high efficiency of EV uploading [4]. On this basis, a Nef^mut^ protein truncated at the amino acid 178 (hereinafter referred to as Nef^mut^PL), hence deprived of 29 C-terminal amino acids, was tested (Figure 1 and Supplementary Figure S1).

**Figure 2:**
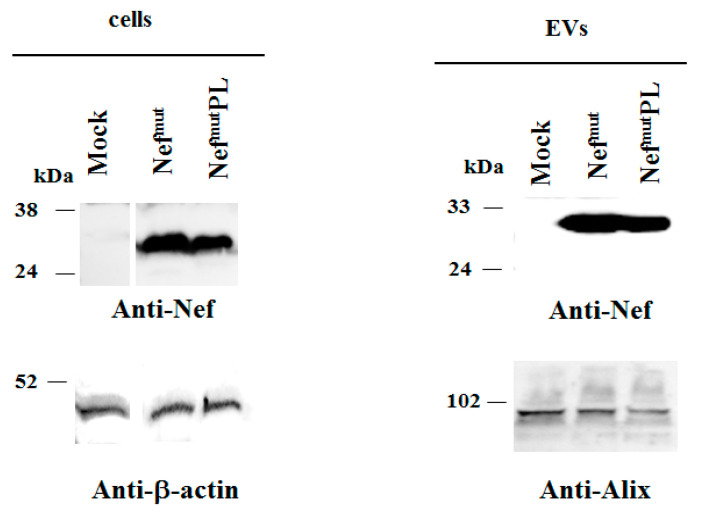
Detection of Nef^mut^PL in transfected cells and EVs. Western blot analysis on 30 µg of lysates of HEK293T cells transfected with DNA vectors expressing either Nef^mut^ or Nef^mut^PL (left panels). Equal volumes of the buffer where purified EVs were resuspended after differential centrifugations of the respective supernatants were also analyzed (right panels). As controls, both cell and EV lysates from mock-transfected cells were included. Polyclonal anti-Nef Abs served to detect Nef^mut^-based products, while β-actin and Alix were revealed as markers for cell lysates and EVs, respectively. Molecular markers are given in kilodaltons (kDa). The results are representative of seven independent experiments.

Both cell accumulation and EV association of Nef^mut^PL compared to the full-length isoform were analyzed. To this aim, western blot analysis on lysates of both transiently transfected HEK293T cells and EVs isolated from respective supernatants were carried out. Representative results are shown in Figure 2 and Supplementary Figure S2 indicated that Nef^mut^ and Nef^mut^PL accumulated into transfected cells at comparable extents, suggesting that the C-terminal truncation did not affect the protein stability. Most important, Nef^mut^PL associated with EVs at levels like those of full-length Nef^mut^, indicating that the presence of the 29 C-terminal amino acids does not influence the uploading efficiency of Nef^mut^ into EVs.

### 3.2. Intracellular Expression and EV-Uploading of HPV16-E6 and -E7 Fused with Nef^mut^PL

**Figure 3:**
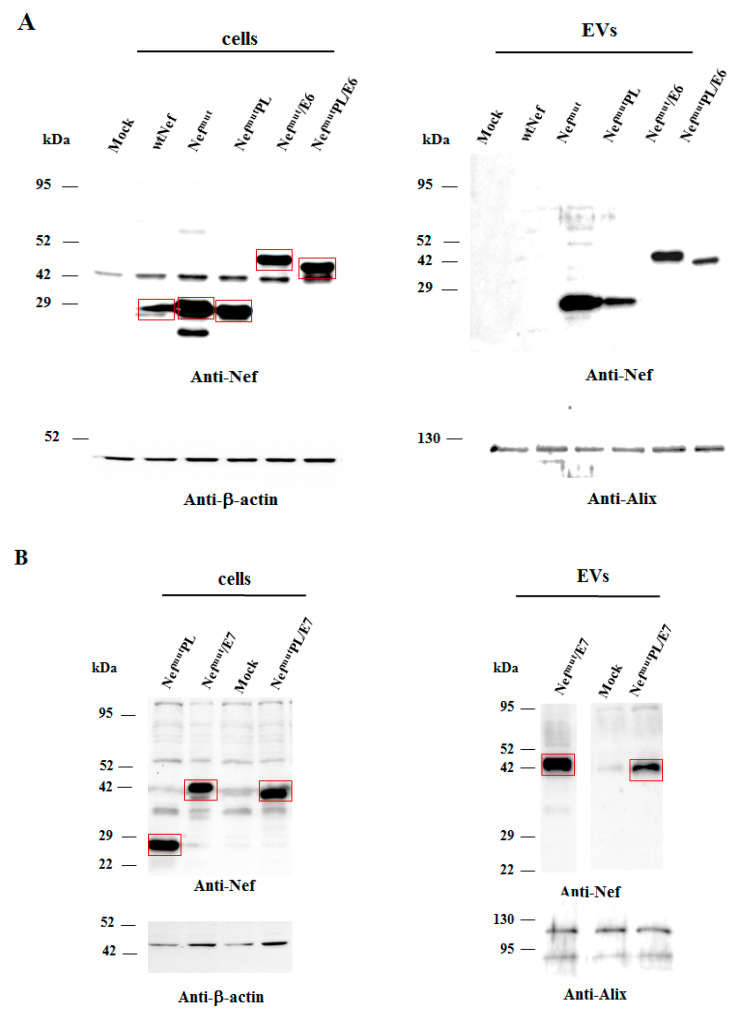
Detection of HPV16-based fusion products in both transfected cells and EVs. (**A**) Western blot analysis on 30 µg of lysates of HEK293T cells transfected with DNA vectors expressing the indicated fusion products (left panel), and equal volumes of the buffer where purified EVs were resuspended after differential centrifugations of respective supernatants (right panel). As a control, conditions from mock-transfected cells, as well as cells transfected with either wt Nef, Nef^mut^, or Nef^mut^PL, were included. The results are representative of three independent experiments. (**B**) Analysis on 30 µg of lysates of HEK293T cells transfected with DNA vectors expressing the indicated products (left panel), and equal volumes of the buffer where purified EVs were resuspended after differential centrifugations of respective supernatants (right panel). As a control, conditions from mock-transfected cells were included. The results are representative of four independent experiments.

Next, we tested the efficiency of Nef^mut^PL to vehiculate foreign proteins fused to it into EVs compared to parental Nef^mut^. We considered both HPV16-E6 and -E7 as foreign antigens since they are uploaded in EVs very efficiently upon fusion with full-length Nef^mut^ [6,10]. The Nef^mut^-related sequences were fused with either HPV16-E6 or -E7 ORFs, which were optimized for translation in eukaryotic cells, and whose protein domains involved in the respective pathogenic effects were inactivated as hereabove described. Steady-state levels in both transfected cells and EVs isolated from the respective supernatants were evaluated by western blot analysis. The E6-based fusion products accumulated in HEK293T transfected cells, as well as in EVs at similar levels whatever the anchoring protein. (Figure 3A and Supplementary Figure S3). Similarly, E7 fused with either Nef^mut^ or Nef^mut^PL was expressed and uploaded in EVs at similar extents (Figure 3B and Supplementary Figure S4).

We concluded that the presence of the C-terminal 29 amino acids of Nef^mu^ is not essential for the efficient EV-uploading of products fused to it.

In both panels, polyclonal anti-Nef Abs served to detect Nef^mut^-based products, while β-actin and Alix were markers for cell lysates and EVs, respectively. Where relevant, specific signals are highlighted. Molecular markers are given in kDa.

### 3.3. Induction of Antigen-Specific Polyfunctional CD8^+^ T Lymphocytes in Mice Immunized with DNA Vectors Expressing Nef^mut^PL/E6 and /E7

The induction of a strong CTL immunity implies the generation of antigen-specific CD8^+^ T lymphocytes co-expressing inflammatory Th-1 cytokines including IFN-γ, IL-2, and TNF-α (i.e., polyfunctional CD8^+^ T lymphocytes). We compared the effectiveness of Nef^mut^ and Nef^mut^/PL in eliciting the antigen-specific CD8^+^ T cell immune response in terms of induction of polyfunctional CD8^+^ T lymphocytes.

**Figure 4:**
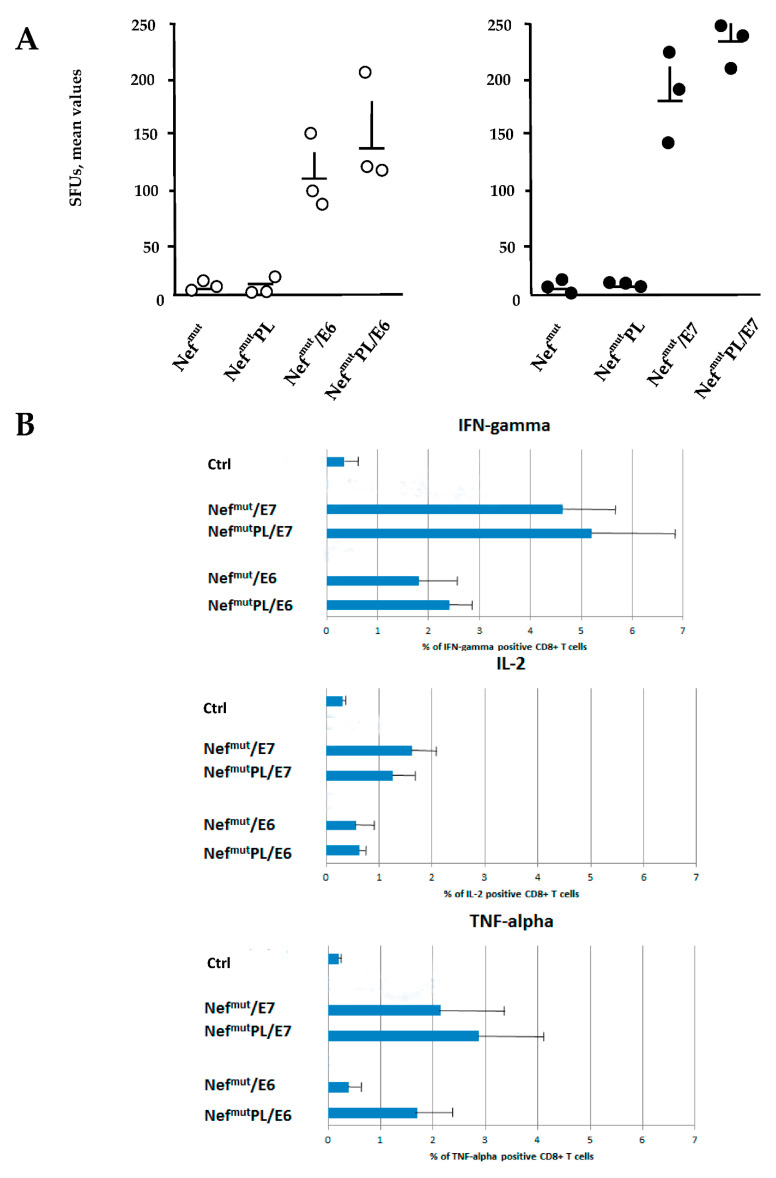

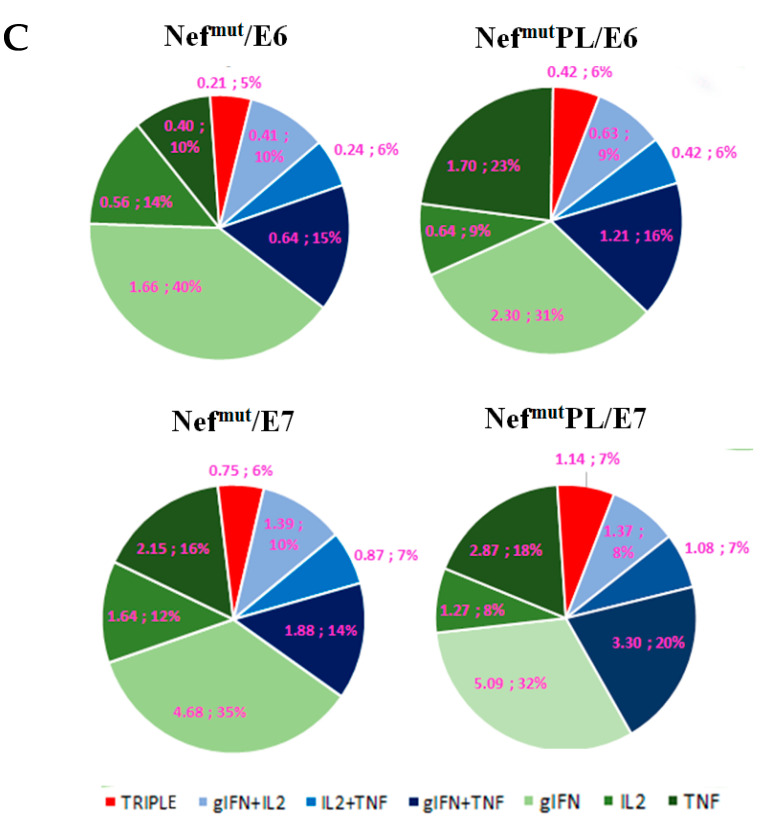
HPV16 E6-and E7-specific CD8^+^ T cell immunity induced in mice after injection of DNA vectors expressing either Nef^mut^ or Nef^mut^PL. (**A**). CD8^+^ T cell immune response in C57 Bl/6 mice inoculated with DNA vectors expressing the indicated fusion products. As a control, mice were inoculated with vectors expressing either Nef^mut^ or Nef^mut^PL. At the time of sacrifice, 2.5 × 10^5^ splenocytes were incubated ON with or without 5 µg/mL of either unrelated (not shown), E6, or E7-specific peptides in triplicate IFN-γ EliSpot microwells. Shown are the number of IFN-γ SFUs/well as mean values of triplicates. Intragroup mean values +SD are also reported. (**B**). IFN-γ, IL-2, and TNF-α expression in CD8^+^ T cells from cultures of splenocytes isolated from each mouse injected with the indicated DNA vectors. Splenocytes were incubated ON with or without 5 µg/mL of either unrelated, E6 or E7-specific peptides in the presence of brefeldin A, and then analyzed by ICS. Shown are the mean values +SD of positive CD8^+^ T cells within total alive CD8^+^ T cells for each cytokine, as calculated after subtraction of values detected in CD8^+^ T cells from cultures treated with the unrelated peptide. Ctrl: mean values from cultures of splenocytes isolated from mice injected with Nef^mut^ and Nef^mut^PL expressing vectors. (**C**). Pie charts indicating the means of both absolute (i.e., over the total of analyzed CD8^+^ T cells) and relative percentages of CD8^+^ T cells expressing cytokine combinations within pools of splenocytes from mice injected with the indicated vectors. Percentages were calculated after subtraction of values detected in homologous cultures treated with unrelated peptides. Values detected with splenocytes from mice injected with control vectors were below the sensitivity threshold of the assay (not shown). The results are representative of two independent experiments.

DNA vectors expressing HPV16-E6 and-E7 fused with either Nef^mut^ or Nef^mut^PL were injected in mice. Fourteen days after the second immunization, IFN-γ EliSpot assays showed that all injected mice developed a well detectable antigen-specific CD8^+^ T cell immunity (Figure 4A), with DNA vectors expressing E7-derivatives inducing stronger immune responses compared to E6-derivatives. We noticed that the levels of CD8^+^ T cell immune response in mice immunized with Nef^mut^PL-based vectors appeared similar to those detected in mice injected with DNA vectors expressing full-length Nef^mut^ (Figure 4A). The immune response in mice injected with the vectors expressing either Nef^mut^ or Nef^mut^PL remained at the background levels.

Single-cytokine ICS analysis showed that the percentages of E6/E7-specific CD8^+^ T lymphocytes expressing either IFN-γ, IL-2, or TNF-α in no cases decreased when the antigen was fused to Nef^mut^PL (Figure 4B). Most importantly, the C-terminal truncation of Nef^mut^ did not affect the generation of antigen-specific, triple positive, polyfunctional CD8^+^ T lymphocytes (Figure 4C).

We concluded that the anti-HPV16-E6 and -E7 immunogenicity remained largely unchanged when the anchoring protein was deleted of 29 C-terminal amino acids.

### 3.4. Intracellular Expression and EV-Uploading of SARS-CoV-2 Antigens Fused with Nef^mut^PL

Very recently, we demonstrated that the Nef^mut^-based vaccine platform could be instrumental to induce strong CD8^+^ T cell immunity against multiple SARS-CoV-2 antigens as detected in both spleens and lung airways [11]. To establish whether these immunogenic properties are preserved also in the presence of the Nef^mut^ C-terminal truncation, we first evaluated the EV-uploading efficiency of the products of fusion of either Nef^mut^ or Nef^mut^PL with S1 and S2 SARS-CoV-2 antigens.

**Figure 5:**
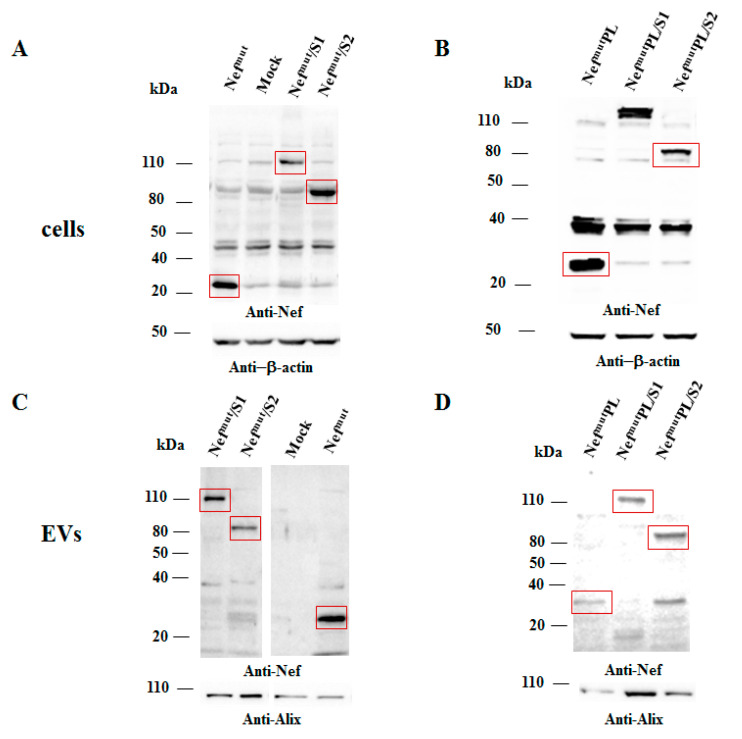
Detection of SARS-CoV-2 S1- and S2-based fusion products in transfected cells and EVs. Western blot analysis on 30 µg of lysates from HEK293T cells transfected with DNA vectors expressing either Nef^mut^ or Nef^mut^PL fused with either SARS-CoV-2 S1 or S2 ORFs (**A**,**B**). Equal volumes of the buffer where purified EVs were resuspended after differential centrifugations of the respective supernatants were also analyzed (**C**,**D**). As a control, conditions from mock-transfected cells, as well as cells transfected with Nef^mut^PL expressing vector, were included. Polyclonal anti-Nef Abs served to detect Nef^mut^-based products, while β-actin and Alix were revealed as markers for cell lysates and EVs, respectively. Where relevant, specific signals are highlighted. Molecular markers are given in kDa. The results are representative of two independent experiments.

Intracellular expression of the fusion products was evaluated by transient transfection in HEK293T cells, and the relative levels of uploading into EVs were scored upon their isolation from the supernatants of transfected cells. Results from western blot assays indicated that S1- and S2-based fusion proteins accumulated in cells at comparable extents whatever the EV-anchoring protein considered (Figure 5 and Supplementary Figure S5). Most important, no evident differences in the levels of EV-uploading of the fusion products were assessed in the presence of a slight decrease in EV uploading of both Nef^mut^/S1 and /S2 compared to Nef^mut^ alone (Figure 5 and Supplementary Figure S5).

We concluded that, as already observed with HPV16 products, the efficiency of EV uploading of SARS-CoV-2 antigens was not influenced by the Nef^mut^ C-terminal truncation.

### 3.5. CD8^+^ T Cell Immunity Induced in Both Spleens and Lungs of Mice Injected with Vectors Expressing Nef^mut^PL-S1 and -S2

**Figure 6:**
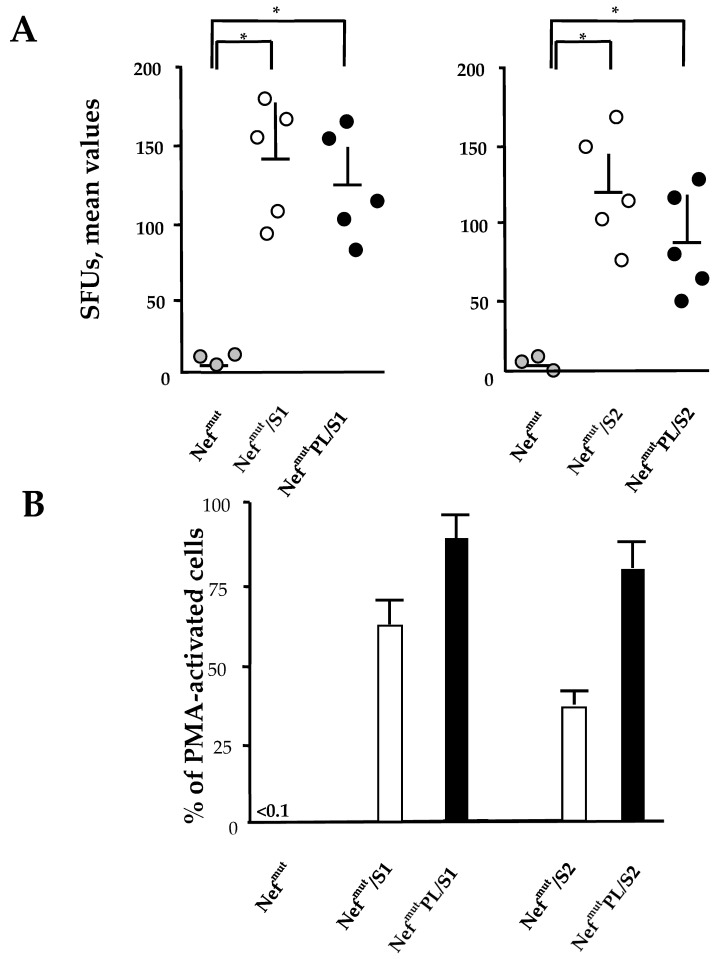
SARS-CoV-2-specific CD8^+^ T cell immunity induced in both spleens and lungs of DNA injected mice. (**A**). CD8^+^ T cell immune response in either C57 Bl/6 (for S1-expressing vector) or Balb/c (for S2-expressing vector) mice inoculated i.m. with DNA vectors expressing either Nef^mut^ or Nef^mut^PL fused with the indicated SARS-CoV-2 antigens. As controls, mice were inoculated with a vector expressing Nef^mut^. At the time of sacrifice, 2.5 × 10^5^ splenocytes were incubated ON with or without 5 µg/mL of either unrelated or SARS-CoV-2-specific peptides in triplicate IFN-γ EliSpot microwells. Shown are the numbers of IFN-γ SFUs/well as mean values of triplicates after subtraction of mean values measured in wells seeded with splenocytes treated with unspecific peptides. Intragroup mean values + SD are also shown. The results are representative of two independent experiments. Statistical significance compared to values obtained with splenocytes injected with Nef^mut^ expressing vectors was determined by the Mann-Whitney U test. * *p* < 0.05. (**B**). Percentages of SFUs+SD detected in IFN-γ EliSpot microwells seeded with 10^5^ cells from pooled BALFs in the presence of SARS-CoV-2-specific peptides compared to PMA plus ionomycin, which generated from 100 to 300 SFUs. Cell samples seeded with unrelated peptides scored at background levels (not shown). The results are representative of four independent experiments.

Both SARS-CoV-2 S1 and S2 were previously shown to induce a strong CD8^+^ T cell immunity in spleens and lung airways when expressed by a DNA vector as products of fusion with Nef^mut^ and injected in mice [11]. On this basis, we compared the CD8^+^ T cell responses following the injection of DNA vectors expressing the two SARS-CoV-2 antigens fused with either Nef^mut^ or Nef^mut^-PL. Fourteen days after the second immunization, splenocytes were isolated from injected mice and cultured overnight in IFN-γ EliSpot microwells. Comparable antigen-specific CD8^+^ T cell activations were observed in splenocytes from mice inoculated with vectors expressing the diverse fusion products, with a slight increase in the case of vectors expressing full-length Nef^mut^ (Figure 6A). On the other hand, the levels of immune responses measured in cells isolated from bronchoalveolar lavage fluids (BALFs) of injected mice appeared similar, with a small increase in cells from mice injected with Nef^mut^PL-based vectors (Figure 6B).

These data strongly supported the idea that the Nef^mut^ C-terminus is dispensable for the induction of SARS-CoV-2 specific immunity in spleens and lung airways, i.e., the district primarily involved in SARS-CoV-2-related pathogenesis. Hence, Nef^mut^PL can be considered an effective and safe alternative to full-length Nef^mut^ for anti-SARS-CoV-2 CTL vaccines based on the technology of endogenously engineered EVs.

## 4. Discussion

The perspective to translate into the clinic the Nef^mut^-based CTL vaccine platform requires the optimization of its safety profile. To this aim, the elimination of unnecessary sequences in the Nef^mut^-based vectors would be of utility. Concerning the DNA vector, we already proved that Nef^mut^-based fusion antigens retain their immunogenic properties when expressed by the pVAX1 vector [11], i.e., a vector where prokaryotic sequences are reduced compared to the more complex pTargeT vector we previously utilized. More advanced, extremely small DNA vectors lacking antibiotic resistance (e.g., minicircle DNA vectors) [30] would represent a feasible alternative for translation into the clinic of the Nef^mut^-based vaccine platform. Regarding the possibility to reduce the sequences required for efficient EV-uploading, we recently demonstrated that the presence of the N-terminal domain with associated post-translational modifications is required [31]. On the other hand, it should be considered that the Nef^mut^ defectiveness relies on the co-existence of two amino acid substitutions at positions 153 and 177. Unpredictable, rare, however, theoretically possible events of back-mutation might occur, especially when the DNA vectors undergo large-scale production and delivery. In the case of unwanted back-mutations, possible anti-cellular effects are expected to be strongly mitigated by the deletion of a significant part of the protein. The 29 amino acid C-terminal region deleted in Nef^mut^PL includes domains involved in both signaling and trafficking functions typical of wt Nef [32]. In this way, possibly back-mutated Nef^mut^PL molecules are expected to lose the most detrimental anti-cellular effects.

The truncation of a C-terminal region of 29 amino acids had no impact on both efficiencies of EV uploading and immunogenicity of diverse fusion products. In some instances, these results can be considered unexpected since the Nef C-terminal region comprises the cholesterol recognizing motif (CRM) (Figure 1) already characterized as an important domain for the association of Nef with the viral envelope [33]. Considering the convergence in the biogenesis of HIV-1 and EVs [34], this domain has the potential to play a role in the interaction of Nef with the nanovesicles released by Nef-expressing cells. The CRM amino acid sequence, i.e., LHPEYYK in SF2-like Nef alleles and LHPEYFK in HXB2-like ones, is well conserved among HIV-1 types B strains, including that from which Nef^mut^ originates, i.e., F12/HIV-1 [35]. We hypothesized that the effects of both N-terminal myristoylation and palmitoylation present in Nef^mut^, together with the stretch of basic amino acids located in alpha helix 1, are prevalent over those induced by CRM in terms of stable interaction with lipid rafts of both plasma and endosomal membranes.

## 5. Conclusions

In conclusion, the use of Nef^mut^PL as an anchoring protein can significantly strengthen the safety profile of the EV-based CTL vaccine platform for new strategies against infectious and tumor diseases.

## Data Availability

The data presented in this study are available on request from the corresponding author. The data are not publicly available due to patent application.

## Supplementary Materials

The following are available online at https://www.mdpi.com/article/10.3390/vaccines9040373/s1, Figure S1: Nef^mut^PL sequence; Figures S2–S5: Western blot raw data.
