# Supplementary material for: The C-Terminal Domain of Nefmut Is Dispensable for the CD8+ T Cell Immunogenicity of In Vivo Engineered Extracellular Vesicles
Source: Vaccines (Basel). 2021 Apr 12;9(4):373. doi: 10.3390/vaccines9040373 (PMC8068889; doi:10.3390/vaccines9040373)
Supplement: Supplementary file 1 [file vaccines-09-00373-s001.pdf]

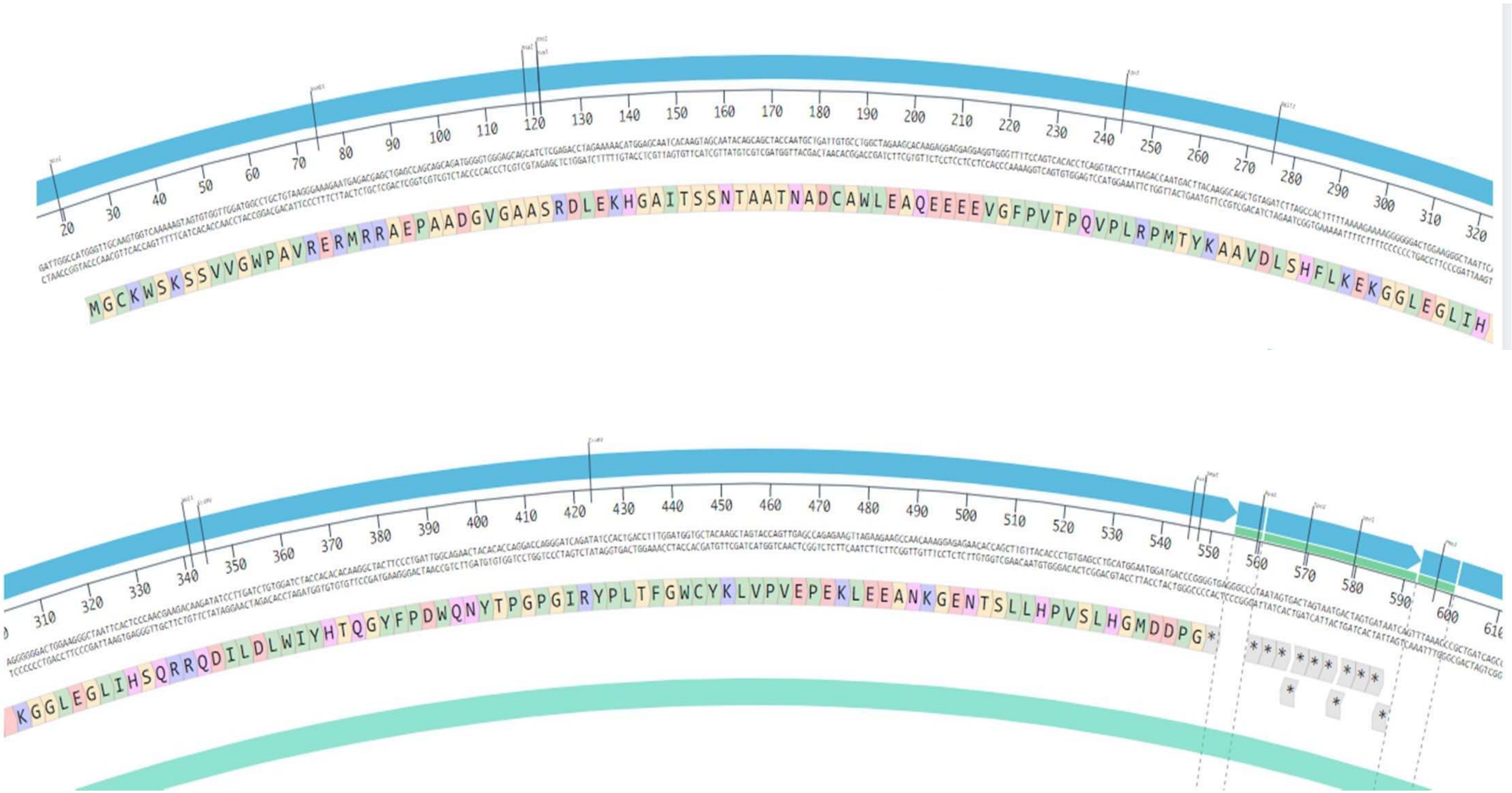

Figure S1. Both nucleotide and amino acid sequence of Nef^{mut}PL.

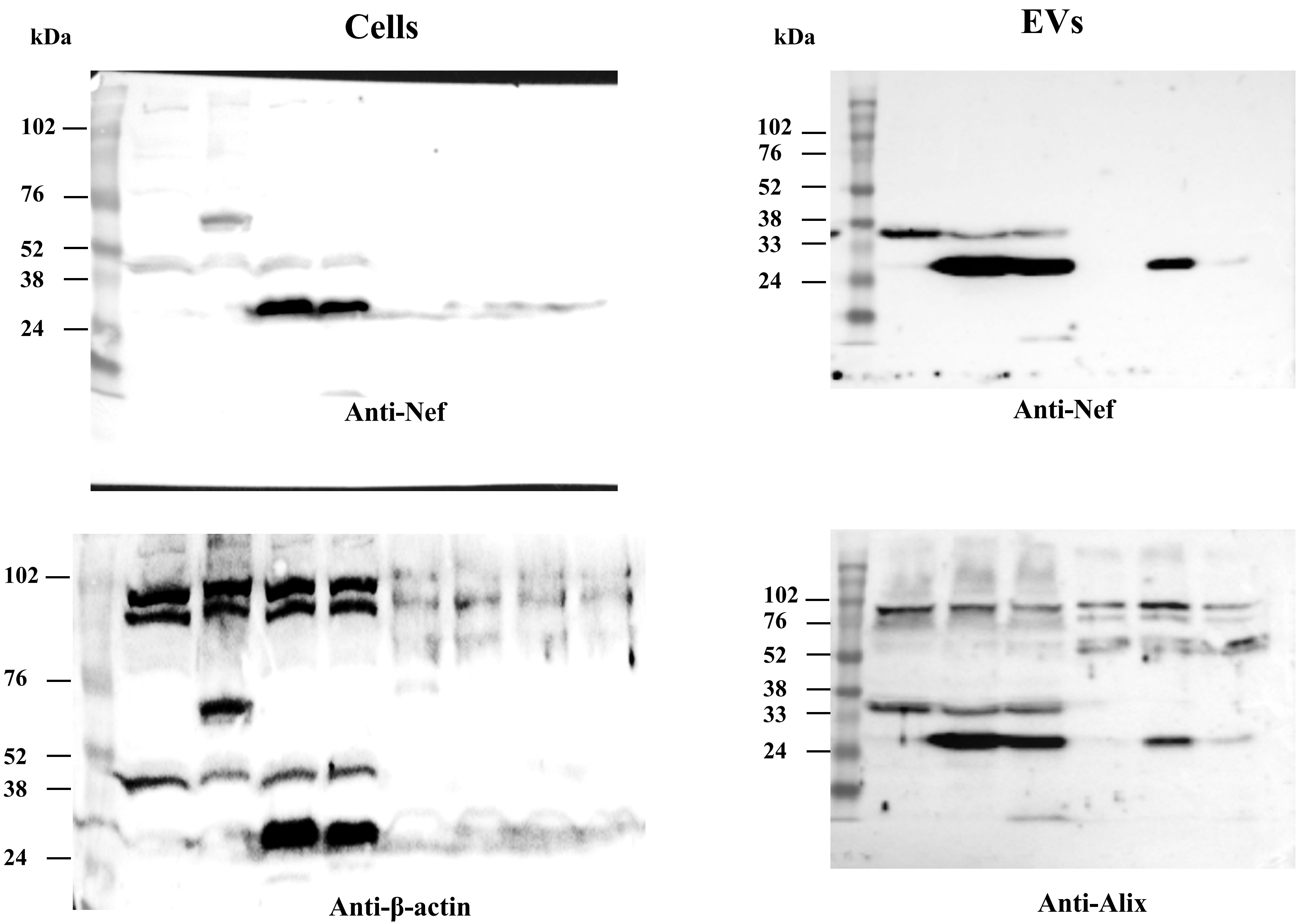

Figure S2. Western blot analysis for the expression of Nef^{mut} and Nef^{mut}PL in transfected HEK293T cells and respective EVs. Raw data.

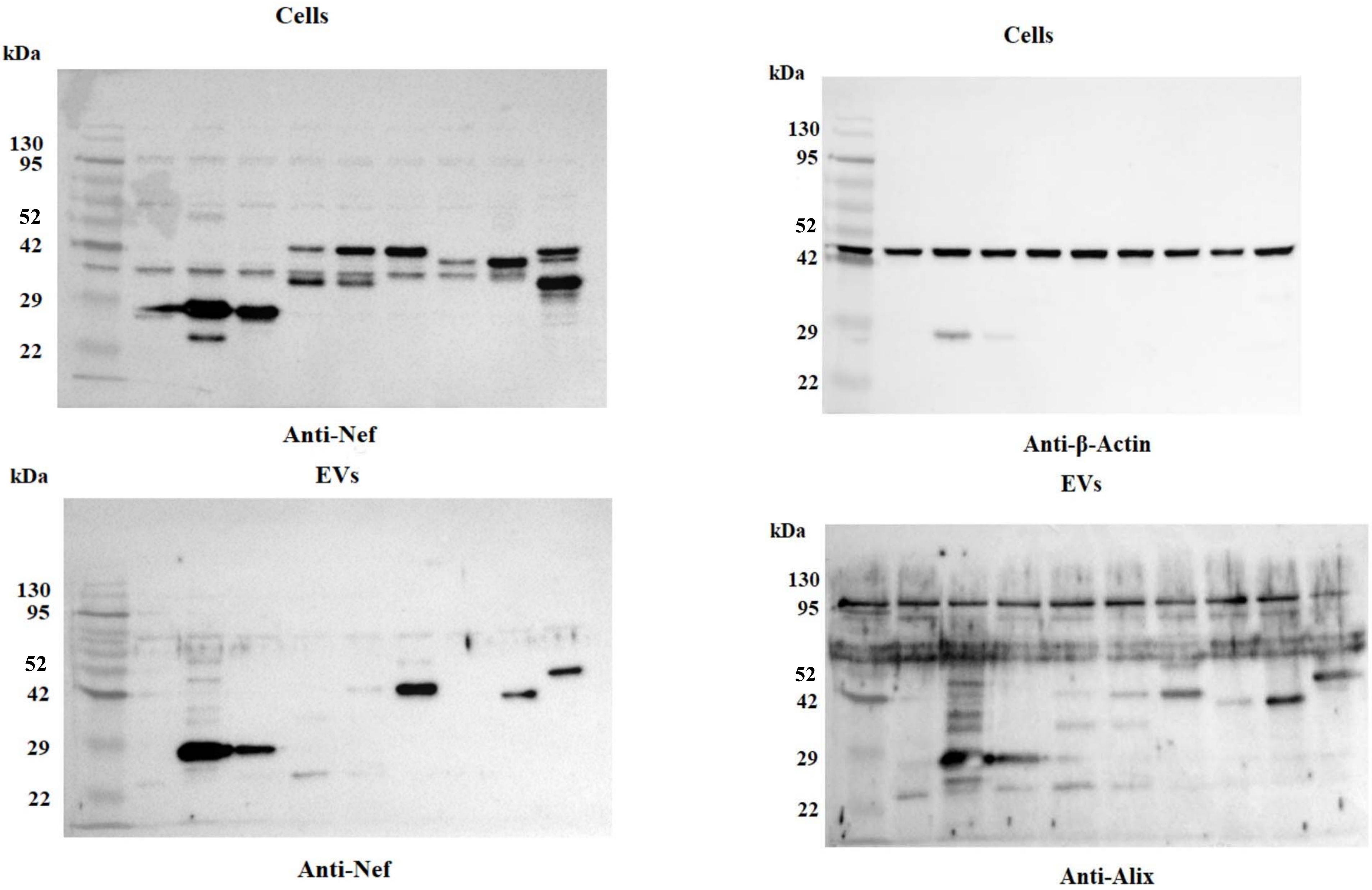

Figure S3. Western blot analysis for the expression of Nef^{mut}/E6 and Nef^{mut}PL/E6 in transfected HEK293T cells and respective EVs. Raw data.

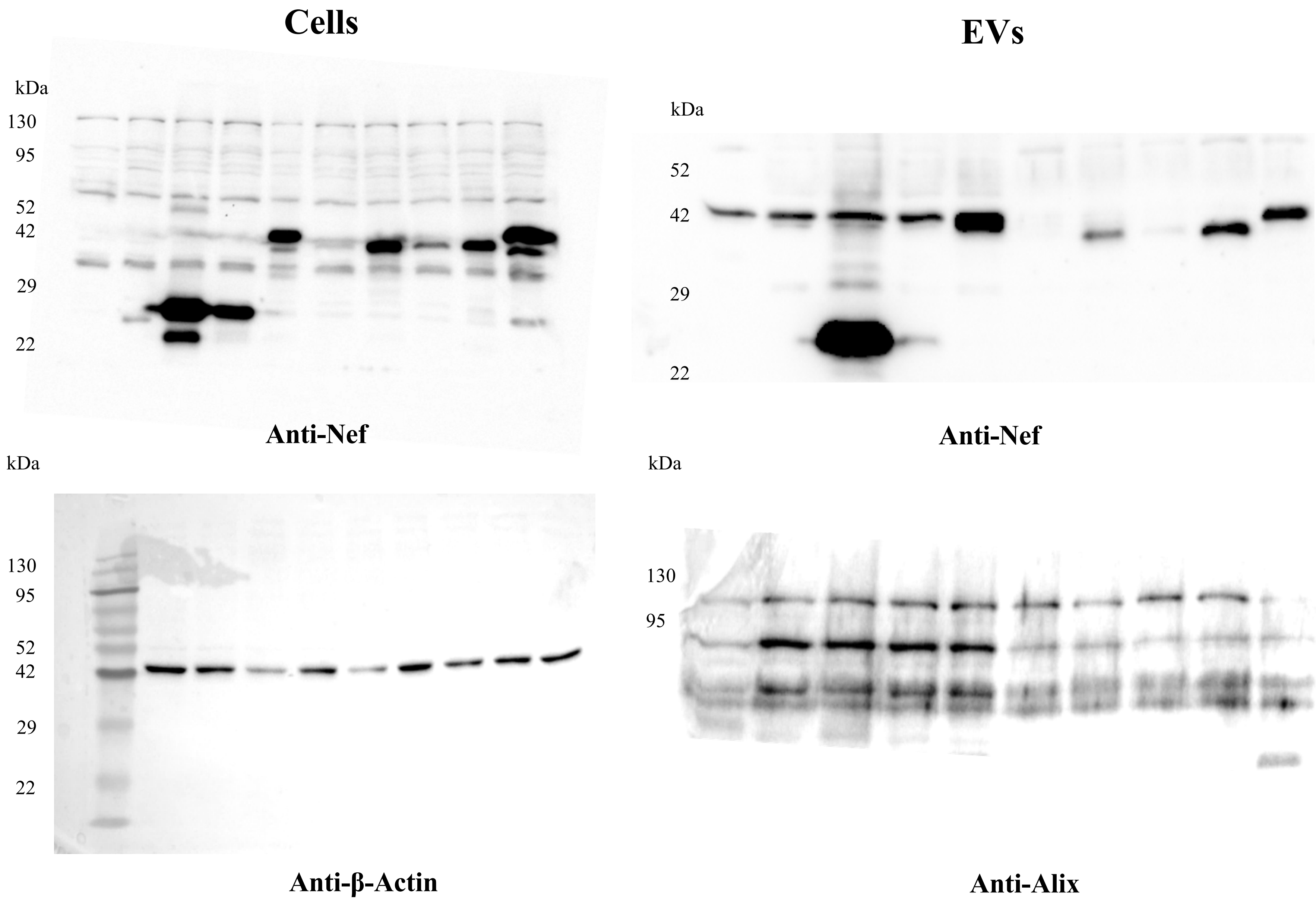

Figure S4. Western blot analysis for the expression of Nef^{mut}/E7 and Nef^{mut}PL/E7 in transfected HEK293T cells and respective EVs. Raw data.

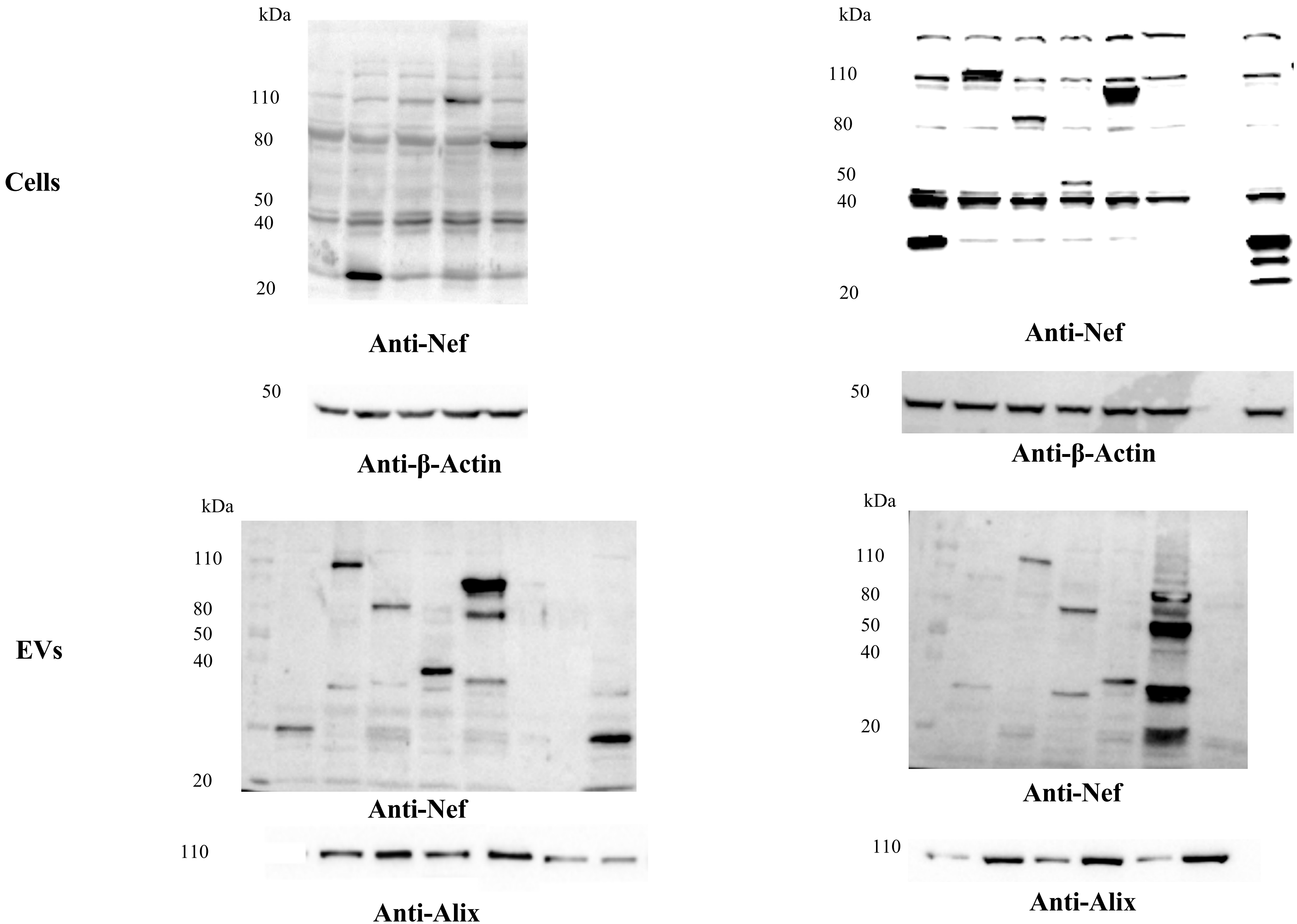

Figure S5. Western blot analysis for the expression of Nef^{mut}/S1, Nef^{mut}/S2 and Nef^{mut}PL/S1 and Nef^{mut}PL/S2 in transfected HEK293T cells and respective EVs. Raw data.
